# Communicative positioning of one's own profession in interprofessional settings

**DOI:** 10.3205/zma001026

**Published:** 2016-04-29

**Authors:** André Posenau, Tim Peters

**Affiliations:** 1 Hochschule für Gesundheit, Department für Angewandte Gesundheitswissenschaften, Bochum, Germany; 2 Ruhr-Universität Bochum, Medizinische Fakultät, Zentrum für Medizinische Lehre, Germany

**Keywords:** Interprofessional education, interprofessional practice, interprofessional collaboration, communication skills, grounded theory, midwifery, nursing, conversation analysis

## Abstract

**Aim: **Interprofessional education (IPE) is taking on increasing importance in our complex healthcare system and receiving ever greater attention in the teaching of health science. The majority of concepts and methods employed in this area are based on normative ideas about interprofessional cooperation and only seldom based on empirical research. This paper is an initial attempt to augment this deductive approach with an inductive perspective for the purpose of subsequently providing empirical support for IPE teaching methods.

**Method: **Drawing on the qualitative approach to linguistic conversation analysis, language-based professional markers are identified on the basis of recorded classroom simulations with nursing and midwifery students; it is assumed that these markers are significant in relevant interprofessional communication processes and, as a result, influence actual collaboration between the health professions. These markers are classified and commented on, and their importance to teaching and practical implementation in interprofessional interaction is emphasized.

**Results: **Students routinely use various professional markers in simulations. However, these occur much less frequently than initially expected, except when marking difference in relation to physicians. At the same time, all the interactions are shaped by pronounced self-presentation among the students, and this comprises a large aspect of the interactions observed here. Profession-specific communication and differentiation processes also appear to be slow in establishing themselves in terms of students delegating tasks or voicing expectations. In addition, the role of “student” has a function that should not be underestimated in these interactions.

**Conclusion:** Professional markers are an essential component of interprofessional communication and are based on numerous, observable linguistic phenomena, of which only a few are presented here. This empirical approach has not yet appeared in the discourse surrounding IPE; however, it is, in the authors’ opinion, not only necessary to compare interprofessional interactions with lived reality, but also to be in a position to operationalize interprofessional practice and ultimately assign it to competency areas. For this reason, further empirical observations and analyses are needed to tighten the still unclear definition of interprofessional communication and to develop empirically founded, measurable criteria for teaching and testing.

## Zusammenfassung

**Zielsetzung: **Interprofessional Education (IPE) ist im sich ausdifferenzierenden Gesundheitssystem von wachsender Bedeutung und nimmt bei der Lehre in Gesundheitsberufen einen immer größeren Raum ein. Der Großteil der Konzepte und Verfahren in diesem Bereich basiert auf normativen Vorstellungen über interprofessionelle Kooperation und ist selten empirisch fundiert. Dieser Beitrag ist ein erster Versuch, die deduktive Herangehensweise mit einer induktiven Perspektive zu ergänzen, um didaktische Konzepte des IPE anschließend empirisch zu untermauern.

**Methodik: **Mithilfe des qualitativen Ansatzes der linguistischen Gesprächsanalyse werden explorativ anhand von im Unterricht aufgezeichneten Simulationen mit Studierenden der Pflege und Hebammenkunde sprachliche Professionsmarker herausgearbeitet, von denen ausgegangen wird, dass sie für relevante interprofessionelle Kommunikationsprozesse von Bedeutung sind und damit die konkrete Zusammenarbeit von Gesundheitsberufen beeinflussen. Diese werden kategorisiert, kommentiert und ihre Bedeutung für die Lehre und die praktische Umsetzung in interprofessionellen Interaktionen herausgestellt.

**Ergebnisse: **Die Studierenden nutzen verschiedene Professionsmarker regelmäßig in den Simulationen. Allerdings treten diese - außer bei der Abgrenzung zu Ärzten - nicht so häufig wie zunächst erwartet auf. Gleichzeitig sind alle Interaktionen von starker Imagearbeit zwischen den Studierenden geprägt, die einen großen Raum in den beobachteten Interaktionen einnimmt. Auch bei der Aufgabendelegation und -beanspruchung scheinen die professionsspezifischen Kommunikations- und Abgrenzungsverfahren sich erst langsam zu etablierenden. Zudem spielt die Studierendenrolle eine nicht zu unterschätzende Funktion in den Interaktionen.

**Schlussfolgerung: **Professionsmarkierungen sind ein wesentlicher Bestandteil interprofessionelle Kommunikation und basieren auf einer (hier nur ausschnitthaft präsentierten) Vielzahl beobachtbarer sprachlicher Phänomene. Dieser empirische Ansatz wurde in der Debatte um IPE bisher nicht aufgegriffen, ist jedoch nach Meinung der Autoren notwendig, um interprofessionelles Handeln mit der gelebten Realität abzugleichen, zu Operationalisieren und schließlich Kompetenzbereichen zuordnen zu können. Daher braucht es weitere empirische Beobachtungen und Analysen, um das teilweise unscharfe Profil interprofessioneller Kommunikation zu schärfen und empirisch fundierte, messbare Kriterien für Lehre und Prüfungen zu entwickeln.

## Authors

Shared first authorship

## 1. Aim

In the discussion about how to define interprofessional settings, communication is repeatedly emphasized as an important competency in connection with the professional roles of those working in health care, and as being significant for successful collaboration [1] [http://www.nklm.de cited 2015 Aug 4]. Yet, no empirical studies currently exist concerning communicative positioning within interprofessional interactions. Frequently, learned roles necessary to education and learning and the resulting mode of interprofessional communication shaped by these roles has been guided by normative ideas and developed in consensus procedures based on experience [2]. In addition, with the increasing relevance of the topic in the curricular context, the issue arises as to how the corresponding competencies can be assessed adequately, since in the authors’ opinion, no empirically founded evaluation scale has been developed. As is the case with practical skills, students cannot be compared with fully trained professionals in the health professions when it comes to interprofessional communication in the curriculum and assessment practices. Therefore, objectives and standards must be adapted to or reformulated for the various levels of education.

To address this issue, concrete empirical data from relevant interactions are necessary to analyze the communication of students’ own professional roles and assign it to competency areas, to then, in turn, be able to teach and assess it in the future on an empirical basis. The aim of this paper is to introduce empirical data gathered from student communication into the current discussion regarding interprofessional communication in education. In the near future, a catalogue will be drafted listing categories for positioning strategies drawn from authentic data, from which empirically based inspiration for teaching and assessing can be derived.

In this paper, we address the issues of how students’ own roles are presented and demarcated in relation to other professions, and which function or communicative task is taken on by these processes in interprofessional settings. Our focus is on the identification and discussion of positioning markers in interprofessional settings involving second-semester students of midwifery and nursing.

In order to fully appreciate the implications for teaching and curriculum, a brief deconstruction of IPE and the object of investigation is necessary, since these theoretical concepts have strongly influenced curricular design and especially the communication setting undergoing analysis here. In essence, the authors subscribe to the definition of CAIPE, the Center for Advancement of Interprofessional Education in the UK, which defines IPE as occurring “when two or more professions learn with, from and about each other to improve collaboration and the quality of care” [3]. In the process of doing this, the material taught should improve collaborative skills, teamwork, understanding the interacting roles, and improving attitudes toward the other professions [4]; all of this can and must be realized primarily through communication.

Furthermore, it must be considered that learning communication skills in this case does not involve learning new material, but rather “unlearning” [5], since students are already familiar with linguistic strategies for controlling conversational processes and with means for expression from various other cooperative settings (group work in school, group presentations, etc.), which, in terms of conversation analysis, do not differ from the communicative methods seen in interprofessional interactions.

## 2. Method

This paper investigates the positioning strategies employed in interprofessional interactions between nurses and midwives as seen in second-semester students at the Hochschule für Gesundheit (hsg) in Bochum, Germany. To improve collaborative skills, students complete six modules on interprofessional interaction, of which the IPE04 module (Interprofessional Communication) is meant to promote pragmatic cooperation between the professions. Theories of communication, communication techniques, conversational features, and psychosocial principles are covered in lectures during the first part, and the material is then practiced and applied during two-day workshops. The module’s core element consists of a simulation and its subsequent evaluation in small groups. To accomplish this, the students were divided into two groups (occupational therapists and physiotherapists, midwives and nurses) and then confronted with appropriate interprofessional patient cases. Due to the narrowed focus on nursing and midwifery, the following illustrations refer only to these two subgroups. It must be noted that all members of the groups investigated here had attained the German Abitur (the school-leaving certificate conferring the right to study at a German university) and thus had been exclusively subject to academic socialization. There were no practitioners with relevant prior experience in these groups.

After receiving the patient case, students were filmed as they brainstormed on how to approach the patient, interacted with the simulated patient, and then conducted an internal follow-up discussion about planned therapy and care. The students then received two hours of feedback in small groups as part of the video analysis to raise their awareness of the interactional processes covered in advance. This approach combines aspects of problem-based learning [6], [7] with simulated patients [8], respectively contact with real patients, with already proven training and feedback methods [9]. These videos also represent the data on which the results presented in this paper are based.

The eight groups investigated here consisted randomly of four to eight nursing and midwifery students. As preparation, the students were given the case information in advance; in this case this was the prenatal care record (Mutterpass) and medical chart of a pregnant patient who had a groin hernia in the third month of pregnancy and suffers from gestational diabetes, as well as an elevated lipoprotein count. Each of these findings had been diagnosed by the attending physician and documented accordingly on the medical records, the disclosure of which the patient had expressly consented to. The students were given the task of advising the patient during a simulated hospital admission and of coming up with appropriate, interprofessional nursing care and additional measures. The students’ initial conferencing took place during one session, contact with the patient and the follow-up discussion during a second one. While all students participated in the initial and follow-up discussions, only two to three students were chosen by the groups to interact with the simulated patient.

The initial discussion, the contact with the patient, and the follow-up discussion were all captured on film, and the resulting 7 hours and 33 minutes of material were then notated in the manner of a score using the transcription program EXMARaLDA [10]. We followed the basic conventions of GAT2 [11], which can at present be described as the standard transcription system for conversational analysis in German linguistics. This system allows observed communication to be presented with differing degrees of detail without having to use coding. We chose to use medium transcript resolution for the data, meaning that along with the content of the spoken language, some relevant, but for practical reasons not all, verbal and nonverbal aspects were documented in writing such as pauses (length in seconds, [1.5]), stressed elements (capitalization, e.g. “NO”), and paraverbal aspects (described in angle brackets, e. g. <<laughing> yes >). All of the transcription conventions are listed in the appendix.

The material was then reviewed separately by the authors and subjected to a detailed analysis using linguistic conversational analysis [12]. When performing the conversational analysis, purely superficially observable linguistic and grammatical phenomena (subjunctives, imperatives or complex technical jargon) were identified at first, and the results were clustered. Based on this, hypotheses were then formulated and explored to identify rules, structures and processes that played a role in the observed communicative context based on frequency, form, placement and function. This qualitative method contains aspects of *grounded theory* [13], discourse analysis [14] and ethnomethodological conversation analysis [15], [16] and has been employed for over 40 years in research on communication in health care [17], [18]. Relevant quality criteria for this kind of qualitative analysis are the quality of the recording and transcription data, the role and consideration of the observer’s paradox, the theoretical saturation and, in particular, the issue of generalizability [12], which will be addressed in more detail in the discussion.

Unfortunately, consideration of the non-verbal aspects was not possible due to the large group of students, the would be necessary numbers of cameras, and the current lack of validated instruments for dealing with such large amounts of data [19]. For this reason, the focus of this study lies on verbal and paraverbal aspects of interprofessional communication.

## 3. Results

The basic results of the analysis are presented here in five subsections that, due to the observed internal and external communicative functions of positive self-presentation, achieving conversational goals, and controlling the structure of the conversation, are viewed as relevant and pedagogically useful. In the transcripts, “St” paired with specific numbers indicates an individual student; the abbreviations P (nursing) and H (midwifery) denote the professional field.

### 3.1. Positioning a profession by naming the profession

**Figure 1:**
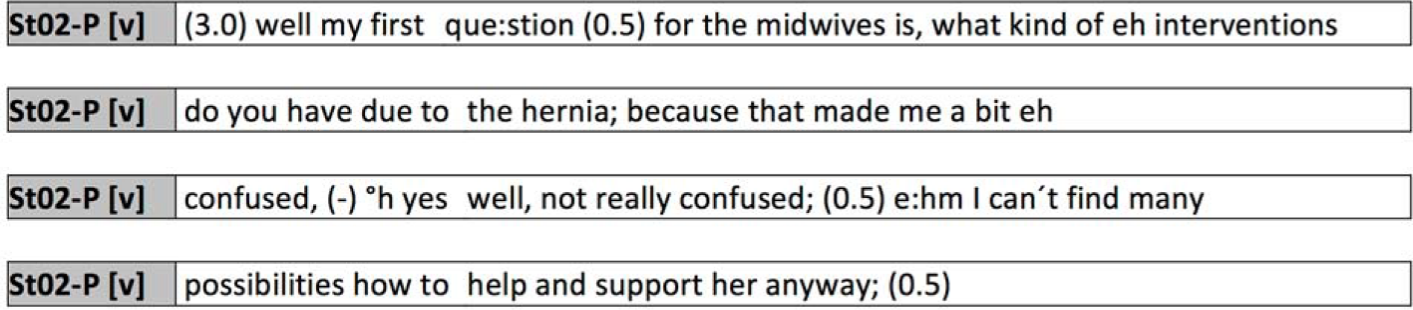
Addressing the individual contributions

A central communicative task which must be carried out at all times during interactions is giving structure to the conversation and addressing the individual contributions to it (see figure 1 (Fig. 1)).

**Figure 2:**
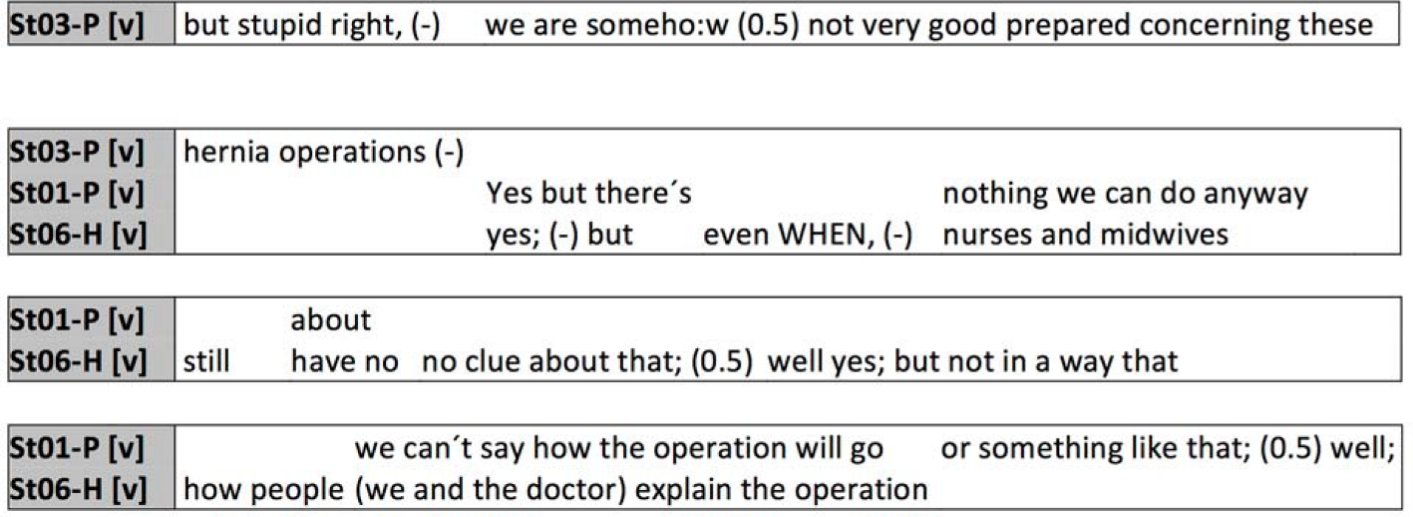
Example responses

In the recorded conversations, the positioning of a profession, as is visible in this example, is frequently accomplished by multiple addresses and the direct naming of the profession. The nursing student does this when addressing a question to the collaborating profession (“well my first que:stion (0.5) for the midwives is,”). By doing this, she addresses only a part of the group and excludes her own profession (nursing) from the obligation to respond. This selection is a competency-based selection through which it is implicitly implied that certain tasks can be accomplished by a certain profession with a higher level of efficiency. It is, however, more a marker giving structure to the spoken exchange, rather than a mostly identity-building one in relation to medical doctors as seen in the following example (see figure 2 (Fig. 2)).

It is frequently observed that when communicative positioning takes place in relation to physicians, this occurs exclusively with a reduction of a profession’s own scope of responsibility and options, as is demonstrated in the statement by St06-H. Positioning in terms of the frequently discussed limitations of one’s own profession and other professions is never used in our corpus as a strategy for taking actions, but rather only as a means of relinquishing competency and passing on responsibility.

In the situation presented here it would also be possible to discuss various options after a formal medical finding has been made in order to react more flexibly to the imminent contact with the patient. This was not seen in any of the interactions, but would very much need to be done in reference to holistic and effective health care. Instead of viewing the physician as an isolated, foreign and potentially superior element, integrating the results and findings of medical practice would allow a high potential for collaboration.

#### 3.2. Positive self-image

**Figure 3:**
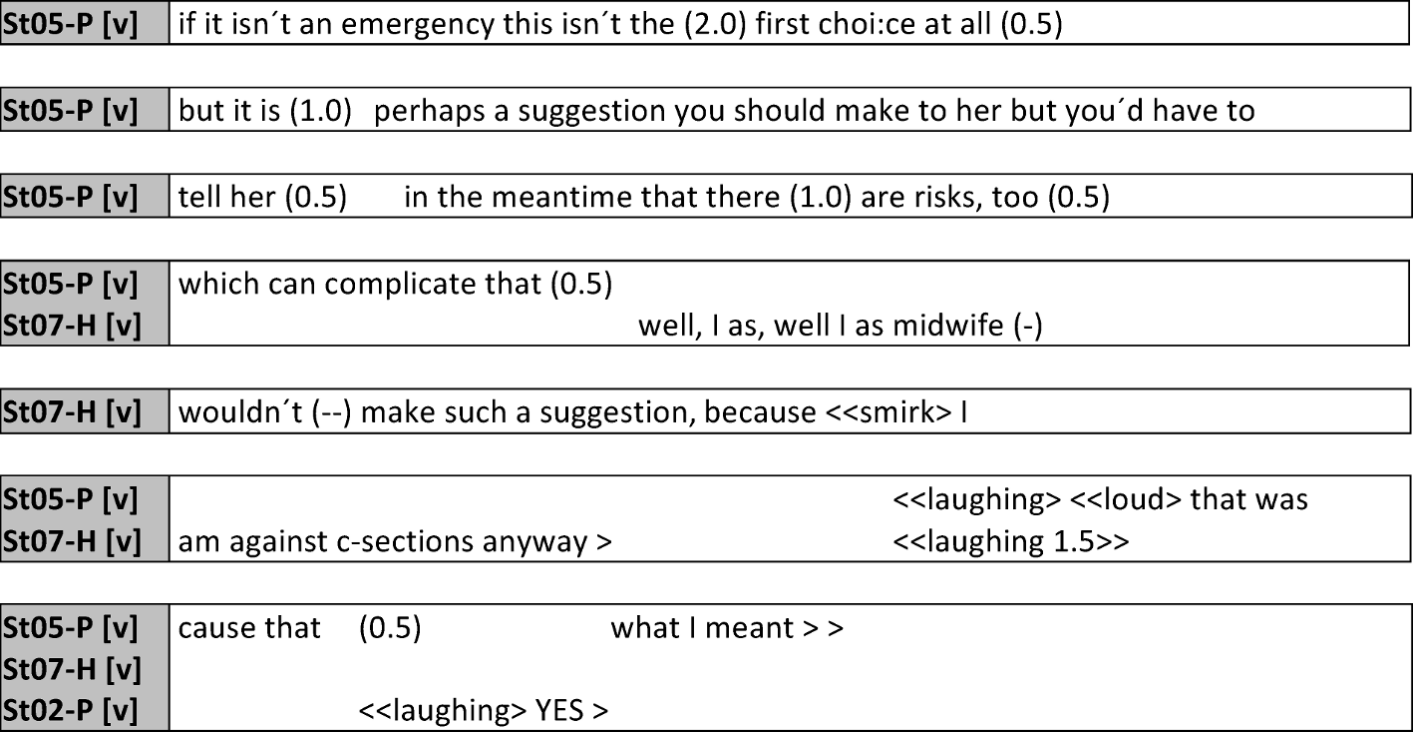
Example responses in conflict situations

Another important and essentially always relevant function is that of self-presentation in interactions, meaning the issue of shaping one’s own identity and the ensuing relationship to others. In respect to this, there are two basic attitudes: a defensive attitude in which one attempts to protect and present one’s own image as competent within the context at hand, and a protective attitude in which this is realized in the other party (in this case, the other students) [20]. Self-presentation becomes particularly tricky in conflict situations, as in the following example, where a nursing student proposes a cesarean section as an option that the midwifery student rejects (see figure 3 (Fig. 3)).

Doubly restricted at first (“if it isn ´t an emergency this isn ´t the (2.0) first choi:ce at all (0.5) but it is (1.0) perhaps a suggestion you should make to her but you ´d have to 

tell her”), nursing student St05-P introduces the cesarean section as a possible course of action into the conversation. The right to speak is then assumed by the midwifery student by naming her own profession (“well, I as, well I as midwife (-) wouldn ´t (--) make such a suggestion,”) and assumes a defensive attitude by “hiding” behind the naming of the functional position since the formulation of a contrary personal opinion could potentially mean a face threatening act for the nursing student. This strategy is, however, rarely followed; most frequently this is expressed by vagueness markers (e.g. adverbs such as “somehow” or subjunctive constructions) and by shared laughter. This method offers a high probability that the relationship will remain unthreatening even in conflict situations. It can also be seen here that with intense self-presentation, communication techniques from other life or work contexts are already known and practiced, and need only be applied in this new surrounding.

#### 3.3. Profession-specific assignment of tasks

**Figure 4:**
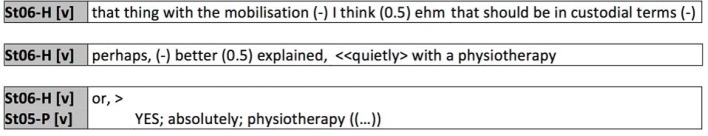
Designation of roles in the conversations

Alongside the more structuring and identity-building devices discussed in 3.1 and 3.2, naming roles in conversation is also used as a means to coordinate efforts (see figure 4 (Fig. 4)).

**Figure 5:**
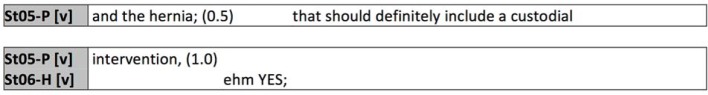
Distribution of tasks by naming the profession as an adjective

Given the clinical picture, midwifery student St06-H focuses on the topic of patient mobilization which falls more clearly under nursing than midwifery. In combination with markers of vagueness (“ehm” and “maybe”), the identity of the other profession is indicated in the German adjectival form (e.g. “maybe best explained from a nursing perspective”) when delegating tasks and responsibilities. Concrete nominal designations (for instance, “that is the responsibility of nursing/midwifery”) are not employed when delegating work. Expression in adjectival form appears to imply vagueness and cautiousness, since the delegation of tasks among equally ranked students has a potential for threat by limiting the freedom to determine one’s own actions [20]. The same is observed when students lay claim to the right to exercise skills or assume tasks (see figure 5 (Fig. 5)).

Early on in the conversation as the clarification of open questions concerning the case are being addressed, St05-P takes the initiative and claims responsibility for handling the hernia using a linguistic device equivalent to the one outlined in the previous example, but conversely to the proposed action. Neither the interventional possibilities the student sees, nor a reason for her assertion are provided as she simply states in German that she sees the need for nursing intervention. Here in the German statements also, in reference to presumed competency only the adjectival, and thus the more cautious, form for assigning concrete tasks is used.

#### 3.4. Transfer of knowledge

**Figure 6:**
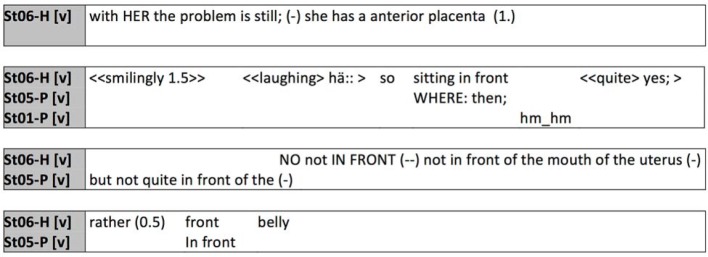
Explanation of the term “anterior placenta”

An essential aspect of interprofessional (and also student) communication is the transfer of knowledge and use of technical terminology. While this will promote precision in expression later on in practice and ensure time-saving when communicating with other professionals [21], for students learning precisely this aspect is the primary focus at present (see figure 6 (Fig. 6)).

**Figure 7:**
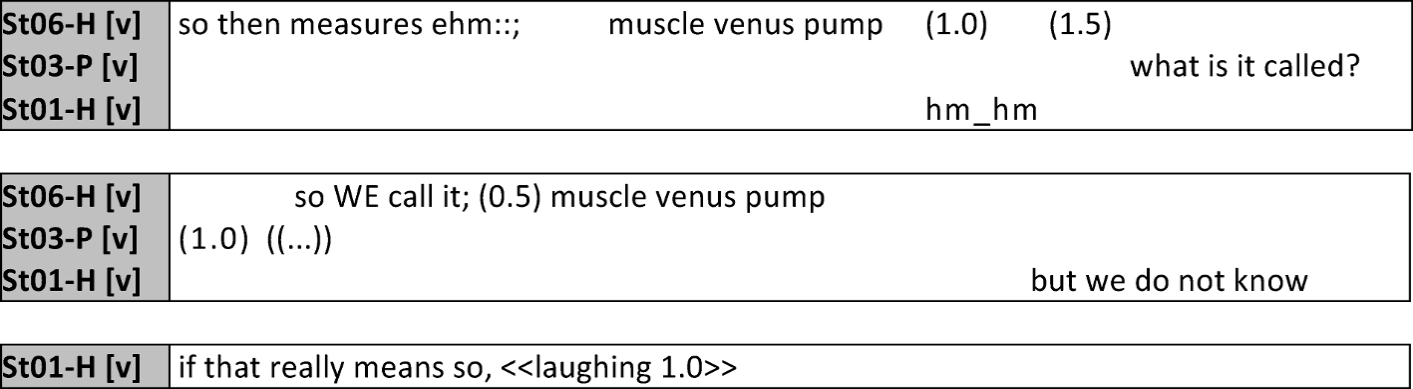
Transfer of knowledge

In this example, the term “anterior placenta” is used by midwifery student St06-H to specify the pregnant patient’s situation. Only after it becomes clear with a pause and a chuckle that no reaction is forthcoming, and the nursing student requests explanation by posing a question, does St06-H elucidate this term. In an interprofessional setting with students the precision and time-saving aspects of technical language do not automatically apply because the term used in this instance was apparently unfamiliar to the nursing students (see figure 7 (Fig. 7)).

In a second example, midwifery student St06-H uses the term “muscle venous pump”, which is confirmed by the other midwife with audible sounds of agreement (“hm_hm”); after a pause nursing student St03-P asks for clarification. The longer length of the pause demonstrates, just as in the first example, the nursing students’ need for detailed explanation and is an indication of insecurity. In her response the midwifery student emphasizes the personal pronoun “we” to stress that this term is used by midwives. A clear delineation between the two groups (midwife vs. nurse takes place through the use of terminology and its explanation. Similar sequences were observed regularly, but not unusually often, in the corpus under investigation here. Technical terms were used by both professions with approximately the same frequency, with those coming from the field of obstetrics being more frequently unfamiliar to the nursing students and leading to clarification and repair sequences more often than in the reverse situation.

#### 3.5. Student-specific addresses

**Figure 8:**
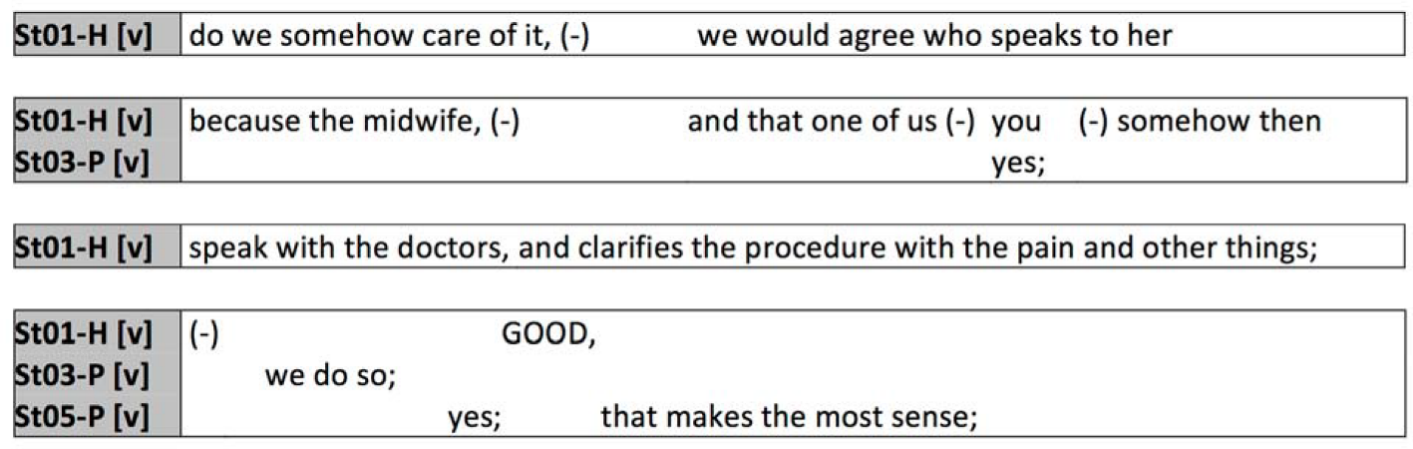
Student-specific addresses in interprofessional communication during a small-group assignment

One result which became visible only when reviewing the empirical material was the influence of the student role. This significantly influenced the interprofessional communication in the group settings (see figure 8 (Fig. 8)).

At the end of the follow-up discussion midwifery student St01-H poses a suggestion that a midwife speak with the patient, and a nurse speak with the physician. Of interest here is the introduction which begins as a question (“do we ...”), makes use of relativization (“somehow”), and finally continues in the subjunctive. The subsequent utterance is also accompanied by many micro-pauses and the use of adverbs irrelevant to the actual content, such as “somehow” and “then”, which are typical of complex expression and imply a weakening of the stated content. The suggestion could also have been formulated much more clearly as a question, or even as a command. The high level of caution exhibited here can even be explained by the fact that the student is assigning the tasks typical to the professions (midwifery students explain the inclusion of a midwife, nursing students speak with the physicians) and very clearly wishes to mark what she says here as a suggestion in order to avert any opposition, avoid any hierarchical relationships, and make a rejection of her proposal possible without incurring damage to any one’s self-image. At the same time, this can also be identified as a typical characteristic of student group work in which suggestions about the delegation of tasks are presumably formulated cautiously and without reference to a hierarchy. Such group dynamics are conceivable in both constellations, and it is not possible to clearly separate them here. Similar situations were observed very frequently in our corpus.

## 4. Discussion

This paper focused on five linguistic devices that play a role in interprofessional communication. It is assumed that even more strategies exist and are relevant, including nonverbal ones. It was not possible to address all of the basic elements of interprofessional communication in this paper due to the data and its scope. With this study, however, it has been possible to initially explore empirically observable phenomena.

In the course of doing this, it became apparent that the linguistic professional markers and the assignment of tasks and competencies played a much smaller role than expected. Although such phenomena were used regularly by students, so that it is possible to speak of established processes, this did not occur with the expected frequency. These were very clearly accompanied by or combined with defensive and protective instances of self-presentation that framed most of the communicative action in critical situations, such as the assignment of tasks or the ethical assessment of findings, such as a potential indication for cesarean section. Only to differentiate from physicians were professional markers and the delegation of tasks clearly observable without much protective self-presentation. The responsibilities of physicians and the options open to them were not productively incorporated into either the conversation or the plans to provide care. Instead, unpopular tasks, insecurities or responsibilities were often assigned away.

At the same time, the extent to which the role of “student” influences interprofessional communication was noticeable; students communicate as groups of students, just as they most likely would on any other group assignment. Pronounced interactions exist between student communication and (inter-) professional communication making a clear classification of the observed phenomena difficult at present. However, the material does suggest that student communication, which is very probably based on earlier modes of school-based communication, serves as a safe base from which students can begin to communicate with other professions and successively develop and establish a new communicative style.

One reason for the results seen in this study is most certainly the early point in the second semester of study when the recording was made. The role of “student” appears to be pronounced and influential, while the professional role has not yet taken on great relevance and must still be internalized. Further studies not only at later study phases, but also involving experienced practitioners are necessary to identify differences and learning curves. In addition, this explorative approach should be continued, but with quantitative methods to better understand the various developmental stages, as well as the realization and relevance of individual communicative acts, including their frequency.

Alongside the need for empirical research mentioned here, and also called for by others [1], a broad discussion must take place about what interprofessional communication means beyond the established models of communication and their techniques, and how this should be measured in future. Only on the basis of hard evidence, unavailable at present, should normative guidelines be formulated and used to define the learning objectives and teaching strategies that will set the stage for teaching and assessing interprofessional communication.

## 5. Attachment

Information on the relevant transcript conventions (see attachment 1 )

## Competing interests

The authors declare that they have no competing interests.

## Supplementary Material

Information on the relevant transcript conventions
