# Supplementary material for: Communicative positioning of one's own profession in interprofessional settings
Source: GMS J Med Educ. 2016 Apr 29;33(2):Doc27. doi: 10.3205/zma001026 (PMC4895853; doi:10.3205/zma001026)
Supplement: Information on the relevant transcript conventions [file JME-33-27-s-001.pdf]

The following lists only the basic conventions that are used in the transcript excerpts presented in this study. They refer to both verbal and paraverbal events. All other conventions not mentioned here can be found in GAT 2 [11].

Aspiration

°h / h°	in- / outbreaths of appr. 0.2-0.5 sec. duration

Pauses

(-)	micro pause, estimated, up to 0.2 sec. duration appr.
(--)	intermediary estimated pause of appr. 0.5-0.8 sec. duration
(0.5)	measured pause of appr. 0.5 sec. duration

Other segmental conventions

and_um	cliticizations within units
eh / oh / uhm	hesitation markers, so-called "filled pauses"
:	lengthening, by about 0.2-0.5 sec.
::	lengthening, by about 0.5-0.8 sec.

Accompanying paraverbal activity

<<laughter 0.5>>	Description of laughter with indication of length
<<laughing> yes, of course >	Laughter particles within speech, with indication of extent

Reception signals

hm / yes / no / nah	monosyllabic tokens
hm_hm / ye_es / no_o	two-syllable tokens

Other conventions

((cough))	non-verbal vocal actions and events
((…))	inability to understand passage with no further information
(such)	possible wording
((incomprehensible 1.5))	incomprehensible passage with indication of length

Accentuation

acCENT	focus accent

Final pitch movements of intonation phrases

?	rising to high
,	rising to mid
-	level
;	falling to mid
.	falling to low

Data set

In compliance with German data protection law, we are not permitted to publish all our data in this publication or to make the videos available online without restrictions. However, we are allowed to share anonymized excerpts, as is the case in section 3. It is also possible to review the materials in the office of the author, Dr. André Posenau, for the purpose of further research or to verify the analyses:

Dr. phil. André Posenau, Vertretungsprofessor

Hochschule für Gesundheit

Gesundheitscampus 6 – 8

44801 Bochum

Office: 3.OG./3341

Email: andre.posenau@hs-gesundheit.de

Telephone: +49 234 77727-650
